# The carotid diaphragm, an often overlooked cause of stroke by cardiologists

**DOI:** 10.1093/jscr/rjac350

**Published:** 2022-07-31

**Authors:** Rim El Mesnaoui, Soumaila Nikiema, Desire Massimbo, Abbes El Mesnaoui

**Affiliations:** Ibn Sina Hospital, Department of Vascular Surgery, Mohammed V University, Rabat, Morocco; Ibn Sina Hospital, Department of Cardiology, Department of Cardiology B, Mohammed V University, Rabat, Morocco; Department of Cardiology, Mohammed V Military Instruction Hospital, Rabat, Morocco; Ibn Sina Hospital, Department of Vascular Surgery, Mohammed V University, Rabat, Morocco

## Abstract

Carotid diaphragm is a rare cause of stroke. Because of its rarity, it remains often undiagnosed. We report the case of four patients who presented a stroke due to carotid diaphragm. The diagnosis was made either by ultrasound Doppler, computed tomography-angiography or angiography. Two of the four patients were managed by carotid stenting and the other two by surgery. The follow-up was normal. Carotid diaphragm stroke is associated with a high risk of recurrence if not well managed. Therefore, the knowledge of this rare entity is necessary.

## INTRODUCTION

The etiology of stroke is not found in one third of cases [1]. The carotid diaphragm is an unrecognized cause of stroke due to its rarity [2]. However, carotid diaphragm stroke is associated with a high risk of recurrence [3]. Therefore, practitioners should be aware of this entity. We report the case of four patients who presented a stroke due carotid diaphragm. Through this work, we present the diagnostic and therapeutic modalities of this pathology.

**Table:** CASE REPORT

Case n°1	Case n°2	Case n°3	Case n°4
• 30-year-old female patient	• 45-year-old patient	• 65-year-old patient, chronic smoker	• 50-year-old female patient without cardiovascular risk factor
• Stroke with right hemiplegia	• Stroke with left hemiparesis	• Stroke with left hemiparesis seen at Day 2	• Persistent headache and dizziness
• Usual etiological work-up negative	• Thrombolysis at Hour 3 with complete recovery	• Usual etiological work-up negative	• Supra-aortic trunks echo Doppler: tight stenosis of the right internal carotid artery
• Supra-aortic trunks and cerebral arteriography: typical aspect of carotid diaphragm	• Supra-aortic trunks echo Doppler + agioscanner: carotid diaphragm with floating thrombus of the right carotid artery(Fig. 1)	• MRI and supra-aortic trunks angiogram: carotid bulb thrombus (Figs 2 and 3)	• Angioscanner: very suggestive of carotid diaphragm
• Treatment by percutaneous carotid stenting	• Operated at Day 8: surgical thrombectomy + thrombendarterectomy by reversal	• Surgery: fresh quasi-occlusive thrombus on carotid diaphragm (Fig. 4)	• Given the stenosing nature of the lesion it is decidet to treat it
• Simple follow-up	• Simple follow-up	• Thrombectomy + carotid Thromboendarterectomy by reversal	• Angioplasty + percutaneous stenting (Fig. 5a–e)
		• Simple follow-up	• Simple follow-up

**Figure 1:**
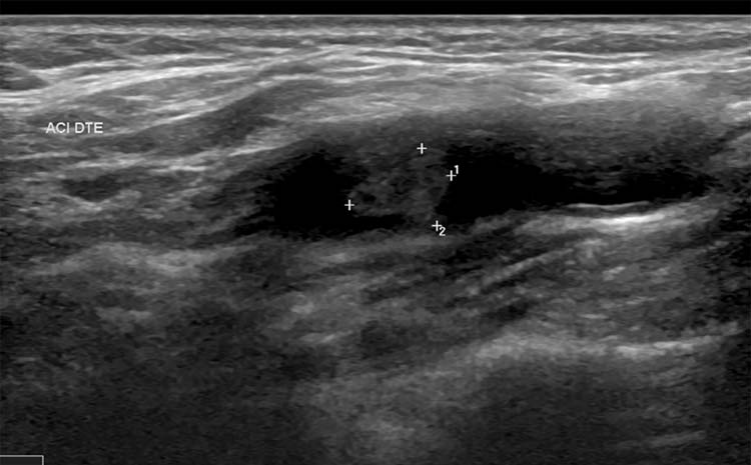
Thrombus of the right carotid artery.

**Figure 2:**
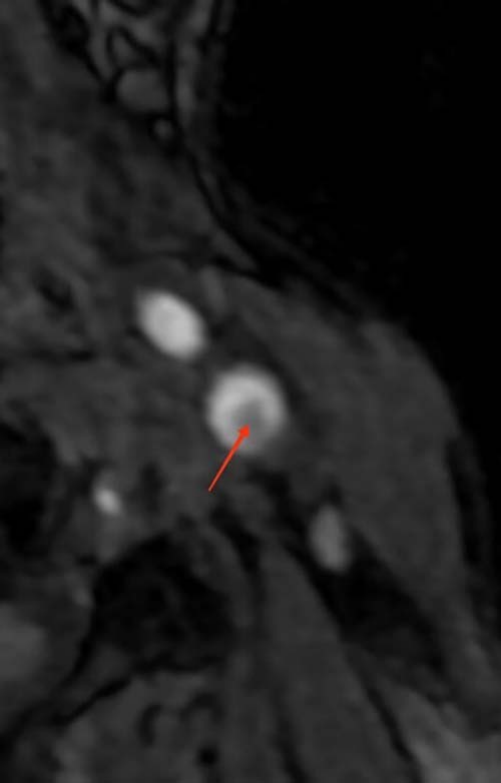
Cross-section showing a bulbar thrombus (MRI).

## DISCUSSION

The carotid diaphragm is a specific, unifocal form of fibromuscular dysplasia (FMD). FMD is a proliferative, non-atheromatous, non-inflammatory process affecting the intima, beyond the sites of atherosclerosis, of the medial and distal portions of medium-sized vessels. The pathogenesis is still unknown, but up to 10% of cases are familial [4]. Microscopic observation shows that it is a proliferation of spindle cell fibrous structures involving the intima layer and to a lesser extent the media layer. Myxoid degeneration has occasionally been described [5]. Adherent thrombus is frequently found on the fibrous intimal thickening [6]. The carotid diaphragm is a non-atheromatous arterial cause of ischaemic stroke, particularly in people under 65 years of age who generally have no cardiovascular risk factors. This abnormality is even more common in the African or Afro-American population [7]. Although the prevalence in the general population is not well known, the incidence of carotid diaphragm was 0.42% in 3600 patients who underwent cerebral arteriography in one study [8]. A female predominance has been reported in several studies, with the age group 40–60 years constituting 60–90% of patients [9]. This malformation can occur in a single artery as well as in several vascular beds; therefore, an appropriate comprehensive diagnostic evaluation is recommended. Indeed, renal arteries are often affected leading to arterial hypertension [4]. Regarding the pathophysiology of stroke, the hemodynamic mechanism has been ruled out. According to the NASCET criteria (The North American symptomatic carotid endarterectomy trial), carotid diaphragm lesions do not generally lead to significant stenosis [10]. The mechanism of stroke is rather thromboembolic. Indeed, a thrombus superimposed on the rostral part of the diaphragm has been described in several imaging and pathological observations [6]. The ‘nest’ formed between the rostral side of the diaphragm and the carotid wall produces flow turbulence with recirculation, blood pooling and stasis that may promote platelet aggregation and thrombus formation [11]. The diagnosis is made by computed tomography (CT) angiography. CT angiography is considered by various authors and physicians to be the method of choice for the diagnosis of carotid diaphragm [5]. It shows either: on sagittal sections: a typical intraluminal filling defect described as a tablet-like membrane or spur that corresponds to a carotid diaphragm lesion [7]; on axial sections: a linear defect that often divides the arterial lumen and thus confers a peculiar appearance called the ‘hamburger’ sign.

**Figure 3:**
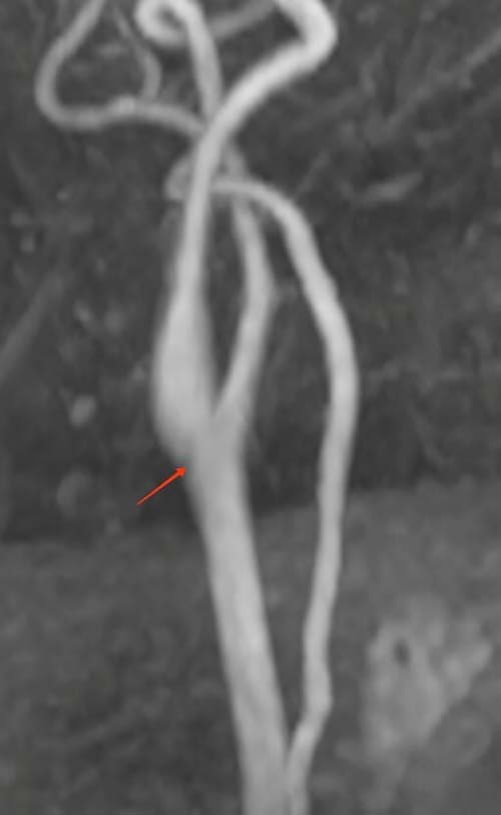
MRI reconstruction image of the left carotid bifurcation showing the carotid diaphragm.

**Figure 4:**
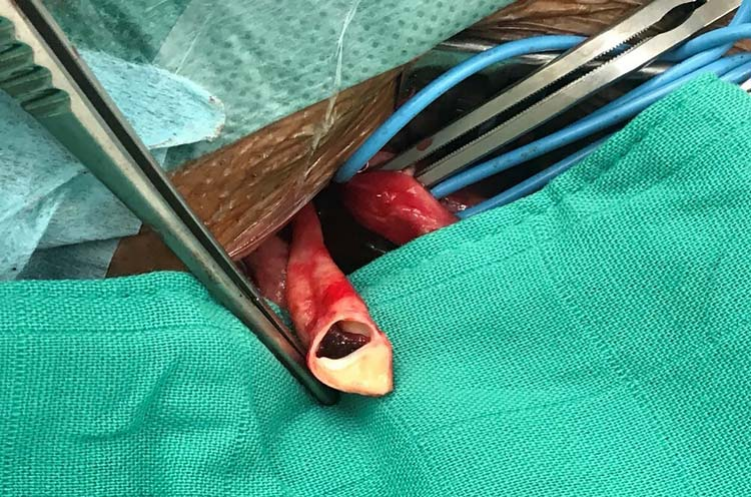
Surgical specimen showing the carotid diaphragm.

**Figure 5:**
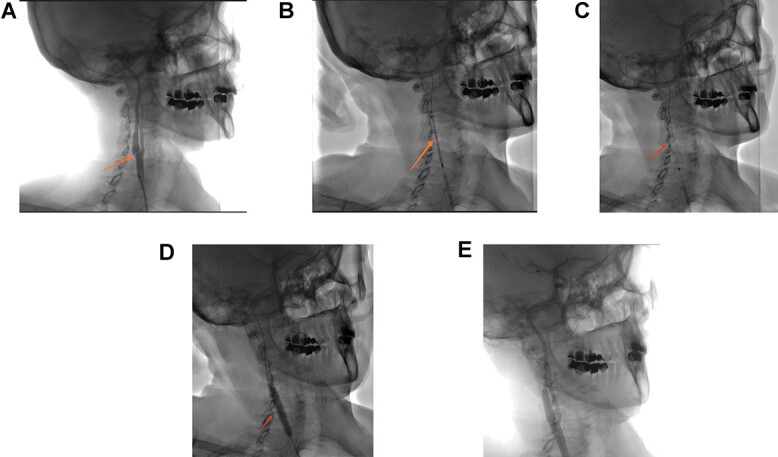
(**A**) Right carotid angiogram image showing the carotid diaphragm (arrow). (**B**) Positioning of the carotid stent. (**C**) Carotid stent release. (**D**) Stent impaction by angioplasty balloon. (**E**) Final angiographic control.

A thrombus found rostral to the carotid diaphragm is described in 12–29% of case series [7].

Doppler of the supra-aortic trunks also helps to approach the diagnosis. The lesion is usually described as an elevated intimal layer followed by a cone or shelf shaped intraluminal defect located on the proximal and posterolateral side of the bulb of the internal carotid artery [6]. It appears moderately hyperechoic and may reveal the presence of thrombi. Ultrasound remains a widely available non-invasive examination, but the results depend on the expertise of the operator. According to some case series, carotid diaphragms are often misdiagnosed or underdiagnosed by the ultrasound doppler [7], which is probably due to their small size, non-limiting aspect and lack of operator awareness of this entity. Multimodal magnetic resonance imaging (MRI) allows the exploration of the carotid wall and could allow, on the basis of further studies, to differentiate the carotid diaphragm from other lesions such as atherosclerotic plaques [6]. Angiography can be considered as the ‘gold standard’ for the diagnosis of the carotid diaphragm because of its high spatial and temporal resolution. It allows analysis of haemodynamic features, many of which have been reported as persistent contrast stasis at the rostral level of the lesion. Management of carotid diaphragm can be surgical, endovascular or medical. Surgical treatment consists of focal excision of the carotid segment where the dysplasia is located followed by artery-arterial anastomosis. It has the advantage of allowing a histopathological examination confirming the dysplastic nature of the lesion. Carotid artery stenting is an endovascular method whose aim is to flatten the defect and avoid blood stagnation. It was initially described in two cases in 2014 [12]. Carotid stenting is performed under dual antiplatelet therapy, usually maintained for three months, followed by long-term monotherapy [13]. Pretreatment with anticoagulant is performed for cases associated with the presence of a thrombus. Compared with surgery, stenting is more recent but proved to be efficient in preventing stroke. A retrospective review of 24 patients admitted to five comprehensive stroke centers conducted by Haussen *et al*. [13] showed that stenting for symptomatic carotid diaphragm appears to be a safe and effective alternative to surgical resection. Medical treatment alone with anti-platelet agents and anticoagulants is associated with a high risk of recurrence.

## CONCLUSION

Carotid diaphragm is a rare cause of stroke. A good knowledge of this entity will help not to overlook it. Ultrasound doppler and CT angiography are non-invasive tools to diagnosing carotid diaphragm. But angiography is the gold standard for the diagnosis. The medical management alone is found to be associated with a high risk of reccurence. Surgery and carotid stenting can be performed to avoid recurrences.

## AUTHORS CONTRIBUTION

All authors have contributed to this work.

## CONFLICT OF INTEREST STATEMENT

None declared.
